# APPLYING THE THERAPEUTIC LANDSCAPE MODEL TO IRISH MEN’S SHEDS

**DOI:** 10.1093/geroni/igad104.2677

**Published:** 2023-12-21

**Authors:** Melinda Heinz, Frank Houghton

**Affiliations:** Upper Iowa University, Fayette, Iowa, United States; Technological University of the Shannon, Limerick, Limerick, Ireland

## Abstract

Gesler’s (1993) introduction of the therapeutic landscape model considers whether environments positively contribute to the wellbeing of users of the space. The model includes both inner/meaning components that consist of 1) natural setting, 2) built environment, 3) sense of place, 4) symbolic landscape and 5) everyday activities and outer/society components of 1) beliefs and philosophies, 2) social relations/inequality and 3) territoriality. The purpose of this study was to explore how environments that older adults are engaged in (e.g., Irish Men’s Sheds) exemplify characteristics found in Gesler’s therapeutic landscape model. Interviews with Men’s Sheds participants aged 65+ (N = 37) were conducted in County Limerick, Ireland. After transcription, Braun and Clarke’s (2020) intuitive 6-step process for thematic analysis was used to interpret the data. Interviewee transcripts revealed that the social relations/inequality component of the therapeutic landscape model was most evident in the narratives. The men explained that it was important to create an inclusive space for all men to feel welcome and valued. For example, a participant said, “Some [members] are living on their own, maybe they’re lonely and they feel kind of unwanted…so, the shed has made a difference to them.” Another participant commented, “Every time someone walks into the gate, you’re enthusiastic that they’re here and you try to welcome them, but then you see what they have to offer. Everybody has something.” Future research should investigate how other environments that older adults engage in, embody components of Gesler’s therapeutic landscape model.

